# Pseudo-hypoaldosteronism secondary to infantile urinary tract infections: role of ultrasound

**DOI:** 10.1186/s13052-022-01203-y

**Published:** 2022-01-24

**Authors:** Noemi Graziano, Carlo Agostoni, Francesca Chiaraviglio, Céline Betti, Arianna Piffer, Mario G. Bianchetti, Gregorio P. Milani

**Affiliations:** 1 Department of Clinical Sciences and Community Health, Università degli Studi di Milano, Milan, Italy; 2 Fondazione IRCCS Ca’ Granda Ospedale Maggiore Policlinico, Pediatric Unit, via della Commenda 9, 20122 Milan, Italy; 3 Family medicine Institute, Faculty of Biomedical Sciences, Università della Svizzera Italiana, Lugano, Switzerland; 4 Fondazione IRCCS Ca’ Granda Ospedale Maggiore Policlinico, Pediatric Radiology Unit, Milan, Italy; 5 Pediatric Institute of Southern Switzerland, Ospedale San Giovanni, Bellinzona, Switzerland

**Keywords:** Hyponatremia, Acidosis, Pyelonephritis, Infection, Sonography, Aldosterone; children; infants

## Abstract

**Background:**

The biochemical hallmarks of transient pseudo-hypoaldosteronism associated with a pyelonephritis include hyponatremia, hyperkalemia, and acidosis. We tested if the kidney-urinary tract ultrasound helps in predicting the diagnosis of overt pseudo-hypoaldosteronism in infants with a pyelonephritis.

**Cases presentation:**

Between 2013 and 2020, we managed 71 previously healthy infants 4 weeks to 24 months of age with a pyelonephritis (42 males and 29 females) and made the biochemical diagnosis of pseudo-hypoaldosteronism in 17 (24%). Infants with and without pseudo-hypoaldosteronism did not significantly differ with respect to the prevalence of kidney-urinary tract ultrasound abnormalities, graded by means of the UTD classification system of urinary tract abnormalities.

**Conclusions:**

Kidney-urinary tract ultrasound is almost routinely obtained in children with a febrile urinary tract infection. Our experience does not support the hypothesis that ultrasound might be relevant for the diagnosis of overt transient pseudo-hypoaldosteronism in babies affected by a urinary tract infection. Our data confirm the assumption that negative studies may be important for advancing clinical practice.

## Introduction

Hyponatremia, hyperkalemia and acidosis are the distinctive biochemical manifestations of hypoaldosteronism and overt pseudo-hypoaldosteronism [1, 2]. In countries with a neonatal screening program for congenital adrenal hyperplasia, many babies who present with the mentioned abnormalities are affected by transient secondary pseudo-hypoaldosteronism associated with a severe urinary tract infection, sometimes associated with a dilating urinary tract malformation [3–6]. Therefore, in these cases, the recommended first diagnostic approach includes urine dipstick and culture testing, together with kidney-urinary tract ultrasound [7].

The differential diagnosis is broad for infants without a urinary tract infection and includes transient secondary pseudohypoaldosteronism associated with a dilating urinary tract malformation, primary pseudohypoaldosteronism (i.e. a primary resistance to aldosterone), hypoaldosteronism, adrenal insufficiency, and medication with drugs such as renin-angiotensin-aldosterone system inhibitors, β-blockers, potassium-sparing diuretics, trimethoprim (mostly combined with sulfamethoxazole) and non-steroidal anti-inflammatory drugs [3–8].

In babies with a urinary tract infection, secondary pseudo-hypoaldosteronism represents a temporary kidney tubular under-responsiveness to aldosterone, which is likely brought about by an inflammatory storm within the kidney [1, 3–6]. Preliminary data also postulate the existence of a genetic predisposition in some cases [2].

We speculated that kidney-urinary tract ultrasound might help in further supporting the diagnosis of overt pseudo-hypoaldosteronism. Hereby we present our experience.

## Patients and methods

Between 2013 and 2020, at the Pediatric Emergency Department of the Fondazione IRCCS Ca′ Granda Ospedale Maggiore Policlinico, Milan, Italy, we managed 71 previously healthy infants (42 males and 29 females) 4 weeks to 24 months of age with: 1. an acute urinary tract infection; 2. features consistent with a pyelonephritis: fever (often associated with poor feeding and regurgitations) and at least two of either C-reactive protein ≥9 mg/L (immunoturbidimetry), blood cell count (automated cell counter) revealing an increased total white cell count or an increased neutrophil band-cell count; 3. assessment of sodium, potassium, chloride, acid-base balance (direct potentiometry), creatinine (picric acid assay) and urea (urease assay) in blood; and 4. a kidney-urinary tract ultrasound performed by a sonographer ≤3 days after diagnosis [9].

We made the diagnosis of overt transient pseudo-hypoaldosteronism in cases with at least two of the following three laboratory abnormalities [9]: hyponatremia (sodium < 135 mmol/L), hyperkalemia (potassium > 5.4 mmol/L), and non-gap metabolic acidosis (pH < 7.38, bicarbonate < 20 mmol/L, and difference between sodium and sum of chloride plus bicarbonate < 11 mmol/L). Hyponatremia was classified as mild-moderate (125–134 mmol/L) or severe (< 125 mmol/L), hyperkalemia as mild-moderate (5.5–7.0 mmol/L) or severe (> 7.0 mmol/L), and metabolic acidosis as mild-moderate (bicarbonate 10–19 mmol/L) or severe (bicarbonate < 10 mmol/L).

The abnormalities detected by means of the kidney-urinary tract ultrasound were classified by means of the UTD classification system of urinary tract dilation [10]: stage 1 (normal urinary tract with anterior posterior renal pelvic diameter 10 to < 15 mm and/or central calyceal dilation); stage 2 (anterior posterior kidney pelvic diameter ≥ 15 mm or peripheral calyceal dilation); stage 3 (additional ureteral dilation, abnormal kidney echogenicity or cysts, or bladder abnormalities regardless of anterior posterior kidney pelvic diameter measurement); and stage 4 (kidney swelling). The remaining cases were classified as normal.

Categorical data are presented as counts and percentages and were analyzed using the Fisher exact test. Continuous data are shown as medians and interquartile ranges and were compared using the Mann-Whitney-Wilcoxon test. Statistical significance was defined by two-sided *P*-values of < 0.05.

## Results

Using the aforementioned biochemical criteria, we made the presumptive diagnosis of secondary pseudo-hypoaldosteronism in 17 febrile children presenting with: hyponatremia, hyperkalemia and acidosis (four infants); hyponatremia and acidosis (eight infants), and hyponatremia and hyperkalemia (in the remaining five infants). Hyponatremia, hyperkalemia and acidosis were never severe (Table 1).

**Table 1:** Clinical and laboratory characteristics of infants without and with laboratory features consistent with the diagnosis of transient pseudo-hypoaldosteronism. Data are presented as frequency or as median and interquartile range

	Pseudo-hypoaldosteronism		
	Without	With	***P***-value
*N*	54	17	
Demographics			
Males: Females	31: 23	11: 6	0.76
Age, months	4.8 [2.2–11]	4.0 [3.3–7.3]	0.93
Blood values			
Hemoglobin, g/L	113 [106–122]	114 [104–122]	0.56
Leukocyte count, 10^9^/L	14.4 [11.6–19.9]	14.6 [11.5–19.8]	0.76
Platelet count, 10^9^/L	415 [319–470]	416 [322–469]	0.48
C-reactive protein, mg/L	30 [11–90]	35 [15–60]	0.42
Sodium, mmol/L	134 [133–136]	131 [129–133]	•
Potassium mmol/L	4.7 [4.3–4.9]	5.5 [4.7–6.0]	•
Bicarbonate, mmol/L	24 [22–26]	19 [18–21]	•
Creatinine, μmol/L	27 [20–33]	28 [25–31]	0.53
Urea, mmol/L	2.8 [2.2–3.5]	2.4 [2.2–3.7]	0.33
Altered kidney-urinary tract ultrasound	7*	4^✙^	0.44

^•^the two study groups were not compared with respect to these parameters, because a statistically significant difference is predictable; * UTD classification stage 2: *N* = 1; stage 3: *N* = 6; ^✙^UTD classification stage 2: *N* = 1; stage 3: *N* = 3

Infants without and with the presumptive diagnosis of pseudo-hypoaldosteronism did not significantly differ with respect to female to male ratio, age, blood cell count, C-reactive protein, creatinine and urea levels. Also, the prevalence of ultrasound abnormalities was similar in subjects without and with pseudo-hypoaldosteronism.

## Discussion

Transient secondary pseudohypoaldosteronism is traditionally considered a very rare condition, which is included in Orphanet with the ORPHAcode 93,164. The present data confirm that electrolyte or acid-base disturbances consistent with the diagnosis of overt transient pseudo-hypoaldosteronism are rather common at the onset of infantile pyelonephritis [1, 6, 8, 9]. On the other hand, our results point out that the kidney-urinary tract ultrasound is usually unaltered and therefore of little, if any, value with respect to the presumptive diagnosis of pseudo-hypoaldosteronism.

Pediatric hospitalists consider that the association between acute pyelonephritis and temporary poor responsiveness to aldosterone is very uncommon and feel comfortable with this diagnosis exclusively if the diagnostic work-up also includes the assessment of urinary electrolytes and even laborious tests such as renin, aldosterone, cortisol and 17-hydroxyprogesteron, whose laboratory turnaround time is generally several days [2, 3, 9]. On the other hand, the literature points out that the vast majority of bacterial infections that involve the kidney parenchyma result in a poor tubular responsiveness to aldosterone with decreased potassium and excessive sodium excretion [5]. In most cases, no electrolyte or acid-base imbalance is demonstrated [5]. In many cases, however, there is a tendency towards mild-moderate hyponatremia, hyperkalemia or acidosis [5, 6, 8, 9]. Finally, in a minority of cases, hyponatremia, hyperkalemia or acidosis may be severe and associated among others with distinctive electrocardiographic abnormalities [1–3].

It is widely acknowledged that acute diarrhea predisposes to acidosis and hypokalemia [11]. On the other hand, vomiting is often followed by alkalosis and hypokalemia [12]. Finally, respiratory tract infections are often linked with isolated hyponatremia [11]. On the contrary, the association between infantile pyelonephritis and poor tubular responsiveness to aldosterone with subsequent tendency to the aforementioned disturbances is less recognized. Wider awareness of this association would result in early identification and treatment.

## Conclusions

Kidney-urinary tract ultrasound is almost routinely obtained in children with a febrile urinary tract infection. This experience does not support the hypothesis that ultrasound might be relevant for the diagnosis of overt transient pseudo-hypoaldosteronism in babies affected by a urinary tract infection. Our data confirm the assumption that negative studies may be important for advancing clinical practice.

It is high time for pediatric hospitalists to overcome their hesitancy in considering transient pseudo-hypoaldosteronism owing to the long-held notion of its rarity. Antimicrobial treatment and especially fluid resuscitation with an isotonic solution rapidly restore the acid-base and electrolyte imbalance, thereby further confirming the diagnosis of transient pseudo-hypoaldosteronism.

## Data Availability

The datasets used and/or analysed during the current study are available from the corresponding author on reasonable request.

## References

[1] Bertini A, Milani GP, Simonetti GD, Fossali EF, Faré PB, Bianchetti MG, Lava SAG (2016). Na^+^, K^+^, Cl^−^, acid-base or H_2_O homeostasis in children with urinary tract infections: a narrative review. Pediatr Nephrol..

[2] Tseng MH, Huang JL, Huang SM, Tsai JD, Wu TW, Fan WL, Ding JJ, Lin SH (2020). Clinical features, genetic background, and outcome in infants with urinary tract infection and type IV renal tubular acidosis. Pediatr Res..

[3] Batlle DC, Arruda JA, Kurtzman NA (1981). Hyperkalemic distal renal tubular acidosis associated with obstructive uropathy. N Engl J Med..

[4] Rodríguez-Soriano J, Vallo A, Oliveros R, Castillo G (1983). Transient pseudohypoaldosteronism secondary to obstructive uropathy in infancy. J Pediatr..

[5] Rodríguez-Soriano J, Vallo A, Quintela MJ, Oliveros R, Ubetagoyena M (1992). Normokalaemic pseudohypoaldosteronism is present in children with acute pyelonephritis. Acta Paediatr..

[6] Watanabe T (2004). Hyponatremia and hyperkalemia in infants with acute pyelonephritis. Pediatr Nephrol..

[7] Conversano E, Romano S, Taddio A, Faletra F, Zanon D, Barbi E, Pennesi M (2021). When salt is needed to grow. Pediatr Nephrol..

[8] Memoli E, Lava SAG, Bianchetti MG, Vianello F, Agostoni C, Milani GP (2020). Prevalence, diagnosis, and management of secondary pseudohypoaldosteronism. Pediatr Nephrol..

[9] Milani GP, Grava A, Bianchetti MG, Lava SAG, Dell’Era L, Teatini T, Fossali EF (2017). Electrolyte and acid-base abnormalities in infants with community-acquired acute pyelonephritis: prospective cross-sectional study. Nephron..

[10] Nguyen HT, Benson CB, Bromley B, Campbell JB, Chow J, Coleman B, Cooper C, Crino J, Darge K, Herndon CD, Odibo AO, Somers MJ, Stein DR (2014). Multidisciplinary consensus on the classification of prenatal and postnatal urinary tract dilation (UTD classification system). J Pediatr Urol..

[11] Mazzoni MB, Milani GP, Bernardi S, Odone L, Rocchi A, D’Angelo EA, Alberzoni M, Agostoni C, Bianchetti MG, Fossali EF (2019). Hyponatremia in infants with community-acquired infections on hospital admission. PLoS ONE..

[12] Mersin SS, Ramelli GP, Laux-End R, Bianchetti MG (1995). Urinary chloride excretion distinguishes between renal and extrarenal metabolic alkalosis. Eur J Pediatr..

